# Probabilistic planning for ligament-balanced TKA—Identification of critical ligament properties

**DOI:** 10.3389/fbioe.2022.930724

**Published:** 2022-11-17

**Authors:** Laura Bartsoen, Matthias G. R. Faes, Roel Wirix-Speetjens, David Moens, Ilse Jonkers, Jos Vander Sloten

**Affiliations:** ^1^ Department of Mechanical Engineering, KU Leuven, Leuven, Belgium; ^2^ Chair for Reliability Engineering, TU Dortmund, Dortmund, Germany; ^3^ Materialise NV, Leuven, Belgium; ^4^ Movement Science Department, KU Leuven, Leuven, Belgium

**Keywords:** total knee arthroplasty, probabilistic planning, ligament balancing, musculoskeletal knee model, ligament properties, surgical precision

## Abstract

Total knee arthroplasty (TKA) failures are often attributed to unbalanced knee ligament loading. The current study aims to develop a probabilistic planning process to optimize implant component positioning that achieves a ligament-balanced TKA. This planning process accounts for both subject-specific uncertainty, in terms of ligament material properties and attachment sites, and surgical precision related to the TKA process typically used in clinical practice. The consequent uncertainty in the implant position parameters is quantified by means of a surrogate model in combination with a Monte Carlo simulation. The samples for the Monte Carlo simulation are generated through Bayesian parameter estimation on the native knee model in such a way that each sample is physiologically relevant. In this way, a subject-specific uncertainty is accounted for. A sensitivity analysis, using the delta-moment-independent sensitivity measure, is performed to identify the most critical ligament parameters. The designed process is capable of estimating the precision with which the targeted ligament-balanced TKA can be realized and converting this into a success probability. This study shows that without additional subject-specific information (e.g., knee kinematic measurements), a global success probability of only 12% is estimated. Furthermore, accurate measurement of reference strains and attachment sites critically improves the success probability of the pre-operative planning process. To allow more precise planning, more accurate identification of these ligament properties is required. This study underlines the relevance of investigating *in vivo* or intraoperative measurement techniques to minimize uncertainty in ligament-balanced pre-operative planning results, particularly prioritizing the measurement of ligament reference strains and attachment sites.

## 1 Introduction

Even though total knee arthroplasty (TKA) is widely accepted to treat end-stage osteoarthritis (OA), a revision rate of 5–10% within 10 years (Lidgren et al., 2004) is reported. Survival analysis, however, underestimates the problem, as 20–30% of patients (Delport et al., 2013) present with persistent pain, joint stiffness, and/or are limited in performing activities of daily living (e.g., going upstairs) (Bourne et al., 2010). Noble et al. (2005) even reported that 52% of TKA subjects have functional limitations. Nam et al. (2014) showed that only 66% of the patients indicated that their knees felt “normal” and 54% had residual symptoms. In 47.4% of the cases, revision surgery was related to joint stiffness, instability, or implant loosening (Sharkey et al., 2014). These failures are most often related to sub-optimal patient-specific implant alignment, resulting in unbalanced loading of the surrounding ligaments.

Pre-operative planning can aid the surgeon in identifying the ideal, patient-specific implant position. When performing this pre-operative plan, surgical precision is a large source of uncertainty as has been shown in Bartsoen et al. (2021). Table 1 shows the precision of three different surgical techniques, namely, conventional surgery (De Vloo et al., 2017), patient-specific guides (De Vloo et al., 2017), and robot-assisted surgery (RAS). The precision of RAS from two different systems is reported, namely, MAKO (Hampp et al., 2019) and TSolution One (Cosendey et al., 2021), where Hampp et al. (2019) did not report on all degrees of freedom, long-plural form = degrees of freedom (DOFs). The precision required to achieve a surgical precision for 90% success probability (*Pr*_90%_) (Bartsoen et al., 2021) is given as well, meaning that this surgical precision leads to a 90% probability of TKA surgery resulting in ligament balancing within the post-TKA safe zone, on the condition that patient-specific ligament properties can accurately be measured. Bartsoen et al. (2021) and the current study consider TKA as balanced when forces are generated in the medial and lateral ligaments throughout the squat motion and ligament strains do not exceed 6%. This zone will be further referred to as the “post-TKA safe zone.”

**TABLE 1:** Surgical precision—according to the literature—is reachable with conventional surgery (De Vloo et al., 2017), patient-specific guides (De Vloo et al., 2017), RAS (MAKO (Hampp et al., 2019) and TSolution One (Cosendey et al., 2021)), and *Pr*_90%_ (Bartsoen et al., 2021). It is to be noted that Hampp et al. (2019) did not report on all DOFs; the non-reported DOFs are indicated with “NA.”

Technique		Conventional	Psg	RAS	*Pr*_90%_
Femur	Medial/lateral (mm)	NA	NA	NA and 0.26	1.18
	Anterior/posterior (mm)	NA	NA	NA and 0.33	0.23
	Proximal/distal (mm)	NA	NA	NA and 0.36	0.23
	Flexion/extension (°)	3.32	2.37	0.45 and 0.5	1.19
	Varus/valgus (°)	1.99	1.47	0.18 and 0.3	0.33
	Internal/external (°)	1.97	2.27	0.30 and 0.5	0.28
Tibia	Medial/lateral (mm)	NA	NA	NA and 0.28	0.88
	Anterior/posterior (mm)	NA	NA	NA and 0.43	0.64
	Proximal/distal (mm)	NA	NA	NA and 0.29	0.18
	Slope (°)	2.28	2.42	0.38 and 1.6	0.85
	Varus/valgus (°)	1.81	1.66	0.32 and 0.4	0.23
	Internal/external (°)	9.0	6.28	NA and 0.73	1.87

Currently, pre-operative planning does not consider soft tissue loading, which may contribute to a non-optimal implant position despite pre-operative planning. The importance of ligament balancing was already highlighted in 1977 by Freeman et al. (1977). However, even today, no clear consensus exists on the best method/surgical technique. Typically, knee ligaments are considered to “mainly” be mechanical joint stabilizers. They, however, have a sensory function that also contributes to joint stabilization (Delport et al., 2013). Qualitatively, TKA is considered ligament balanced when the ligaments are appropriately tensioned to provide passive stability without inducing stiffness or pain or limiting motion. The difficulty is, however, to quantify “appropriately tensioned.” Although a few studies (Kuster et al., 2004; Delport et al., 2015; Twiggs et al., 2018) identified a set of quantifiable requirements resulting in a positive outcome, no study has so far identified a conclusive safe zone.

The incorporation of a computational knee model that generates a precise estimation of tibio-femoral (TF) kinematics and consequent ligament strains for each individual patient could be a dedicated approach to account for ligament balancing in pre-operative planning. Most published knee models are rigid-body musculoskeletal models (Smith et al., 2016; Vanheule et al., 2017) and finite element models (Beidokhti et al., 2017). When introducing such a model-based simulation step, the planning process based on a computational knee model introduces uncertainty on several subject-specific parameters (e.g., ligament material properties) that are currently not identifiable in clinical practice in addition to the previously discussed uncertainty introduced by the surgical precision. Not only in rigid-body models but also in finite element models, the ligaments are typically simplified as line elements. The force–strain behavior of such an element can then be described as tension-only with a linear relation between force and strain presenting a quadratic toe region (see Supplementary Material for detailed implementation). This material model requires two subject-specific parameters, namely, the linear stiffness *k* and the reference strain *ϵ*_*r*_. Measurements of these material properties are generally scarce. Some studies (Trent et al., 1976; Woo et al., 1991; Sugita and Amis, 2001; LaPrade et al., 2005; Robinson et al., 2005; Chandrashekar et al., 2006) attempted measuring linear stiffness. From the studies that are available, it can be seen that inter-subject variability is large. In addition, almost no or very limited information on the reference strain is available. Bartsoen et al. (2023) estimated a collection of sets of ligament properties while accounting for physiologically relevant ligament strains, based on *in vitro* experimental squatting data of a knee rig experiment, which typically suffers from measurement errors. The study failed to identify a narrow range of ligament properties applicable to multiple tested specimens. The authors concluded that accounting for uncertainty in each individual ligament property, independent of the other properties, would overestimate surgical outcome uncertainty as a reference strain, and attachment points are highly correlated. They suggested representing uncertainty as a subject-specific collection of sets of ligament properties.

*In vivo* measurement techniques to measure ligament properties not only are emerging (Slane et al., 2017; Pedersen et al., 2019) but also impose specific challenges. For most modeling approaches, it is, however, unknown how the uncertainty of these input parameters affects the simulation results. Such an analysis is, however, highly relevant to evaluate if it is even worthwhile investing in developing *in vivo* measurement methods to identify ligament parameters for application in musculoskeletal knee models (MSKMs) for pre-operative TKA planning. A few studies have investigated the effect of ligament properties on simulated TF kinematics and contact forces (Smith et al., 2016; Beidokhti et al., 2017; Pianigiani et al., 2017). They reported an important influence of the ligament material properties on the TF contact forces. None of these studies, however, investigated the effect of ligament properties on the planned implant position. Such an analysis would be needed to assess if introducing extra measurements obtained based on newly developed measurement techniques needs to be included in the computational knee model for pre-operative planning of TKA.

The current study aims to design and evaluate a planning process for ligament-balanced TKA that accounts for uncertainty in ligament material properties, attachment sites, and surgical precision. The uncertainty in the planned implant position is quantified and reported with a success probability of the ligament-balanced TKA. The uncertainty in the ligament material properties and attachment sites is quantified through a subject-specific collection of sets of ligament properties. In addition, a sensitivity analysis (SA) identifies the most critical ligament properties of the success probability.

## 2 Materials and methods

**FIGURE 1:**
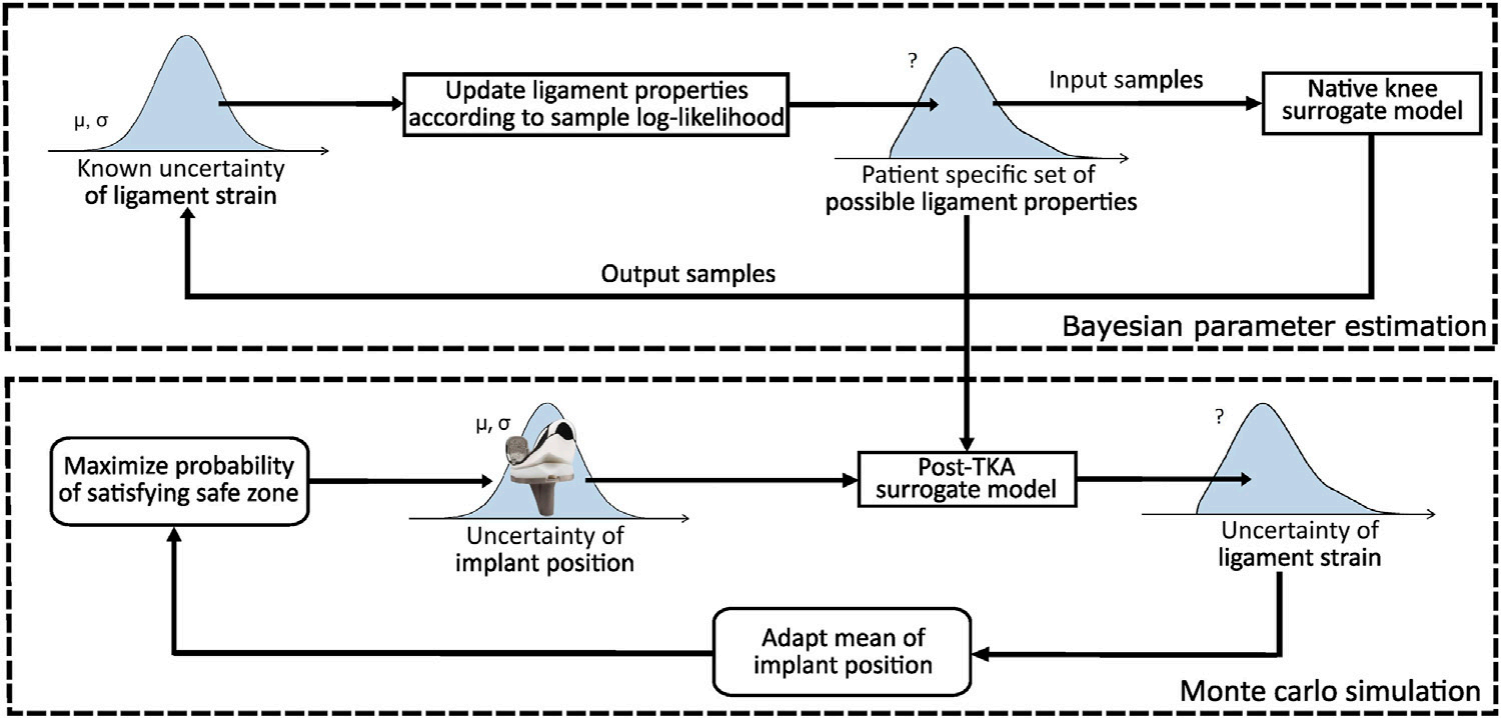
Overview of the probabilistic planning process for TKA.

An overview of the planning process is illustrated in Figure 1. The figure consists of two parts. The top part illustrates how subject-specific uncertainty (Section 2.2) in the ligament properties is quantified, whereas the bottom part optimizes the implant position such that the success probability is maximized. The reader should note that the described success probability indicates the probability of success when the proposed plan is rigorously executed without further adjustments to the implant position and/or ligament releases based on the surgeon’s expertise. In short, the top part illustrates that the subject-specific uncertainty is quantified by identifying a collection of sets (further referred to as “set family”) of ligament properties satisfying a native safe zone.

To generate a set family of matching input parameters, Bayesian parameter estimation (BPE) can be applied given a statistical distribution of the output parameters satisfying the native safe zone. To this end, given the physiologically relevant ligament strains, a set family of subject-specific ligament properties can then be established. To ensure a feasible computational cost, BPE is applied to a surrogate model of the MSKM, simulating a squat motion of the native knee joint. This set family is used to quantify the uncertainty in the planned ligament-balanced implant position parameters. To achieve this, the mean implant position is optimized in order to maximize the global success probability (GSP) (Section 2.4) on a ligament-balanced outcome.

The bottom part of Figure 1 illustrates the optimization of the implant position. Two surgical scenarios are evaluated, namely, RAS (Cosendey et al., 2021) and *Pr*_90%_ (Bartsoen et al., 2021), as well as three different set families are used for describing ligament uncertainty. To ensure a feasible computational cost, this method is executed by training a surrogate model of the MSKM (Section 2.1) simulating a squat motion of the post-TKA knee joint. As a surrogate model, an artificial neural network (ANN) (Section 2.3) is used in order to design an optimization process with a feasible computational time. A sensitivity analysis (SA) (Section 2.5) is performed to identify the most critical ligament properties. More methodological details are provided in the subsequent sections.

### 2.1 Knee model

The knee model is a rigid body MSKM, implemented into the AnyBody Modeling System 7.3.0 (AnyBody Technology A/S, Aalborg, Denmark). The model is based on the knee model described by Vanheule et al. (2017). The implant system is a posterior-stabilized (PS) system (Performance, Biomet Inc., Warsaw, IN, United States). A squat motion from 20° to 120° of flexion is simulated. The model is validated using seven cadaveric specimens, to which a squat motion is applied using a dynamic knee simulator system. The boundary conditions of the dynamic knee simulator are based on the study of Victor et al. (2009a). The authors validated the simulated native and replaced kinematics. The hip joint is allowed to slide vertically and flex/extend. The ankle joint is allowed to rotate along all DOFs and to translate mediolaterally. To each of the hamstring attachments, a force of 50 N is exerted. A variable force is exerted on the quadriceps such that a vertical ankle force of 111 N is achieved. During the simulation, similar DOFs and forces as in the experiment are applied to the knee model. A full description of the MSKM simulation pipeline is presented in the previously published work by our group (Vanheule et al., 2017).

In short, the secondary knee kinematics are computed using force-dependent kinematics (FDK) (Skipper Andersen et al., 2017). This means that eleven DOFs are computed with FDK, five of the TF joints (medial/lateral (M/L), anterior/posterior (A/P), proximal/distal (P/D), varus/valgus (V/V), and internal/external (I/E)) and six of the patellofemoral (PF) joint. This is in contrast with the original model of Vanheule et al. (2017), where the patella tendon length was kept constant, reducing the number of DOFs calculated by FDK to five. FDK is implemented in the AnyBody Modeling System (AnyBody Technology A/S, Aalborg, Denmark).

The model is made as subject-specific as would currently be feasible in a clinical setting. More specifically, the bone geometry and cartilage geometry are segmented from MR images using Mimics 17.0 (Materialise N.V., Leuven, Belgium). As the ligament attachment sites are not clearly visible on MRI, these are initially estimated based on the literature and then perturbed in the parameter estimation. The medial collateral ligament (MCL), lateral collateral ligament (LCL), anterolateral ligament (ALL), popliteofibular ligament (PFL), posterior capsule (PC), anterior cruciate ligament (ACL) with an anteromedial and posterolateral bundle, and posterior cruciate ligament (PCL) with an anterolateral and posteromedial bundle are modeled. Details on the implementation of the ligament material model can be found in the Supplementary Material. The PF ligaments are modeled as spring elements with linear elastic properties between the patella and femur to reduce the computation time.

### 2.2 Subject-specific uncertainty

The subject-specific set family of ligament properties is defined using BPE. BPE allows generating a set family of input parameters based on a given statistical distribution of the output parameters. The native safe zone describes a set of requirements for the model outputs (ligament strains and TF kinematics) that match the behavior of a native knee joint. Through BPE, a set family of knee model input parameters is collected that result in outputs within the requirements. As no consensus exists on the quantification of physiologically relevant knee ligament strains, the current study compares three different set families of ligament properties: 1) the first native safe zone solely accounts for a damage criterion. As ligament damage is unlikely during a squat movement of a healthy knee, ligament property sets that lead to a maximal strain exceeding 6% are excluded from the set family, as Provenzano et al. (2002) and Guo et al. (2015) indeed indicated that ligament damage occurs from this strain level onwards. As no preference for a specific strain is required, a uniform posterior distribution is associated with the maximal ligament strain between the physiological ranges of −2% and 6%. 2) A second native safe zone accounts for damage and stability. Stability is ensured when forces are generated in the medial (deepMCL and supMCL), lateral (LCL, ALL, and PFL), and central ligaments (ACL and PCL) throughout the squat motion. This is based on the study of Victor et al. (2009b) and Delport et al. (2015) that showed that in the native knee, the MCL remains isometric throughout flexion and the LCL stays isometric throughout early and mid-flexion, while tension decreases toward deep flexion. Likewise, Harner et al. (1995) showed the engagement of the ACL in early to mid-flexion, while the PCL ensures anterior/posterior stability from mid to deep flexion. This constraint is quantified as the maximal strain of the medial, lateral, and central ligaments being greater than 0% throughout the squat motion. Similar to the damage constraint, there is no preference for a specific strain. Consequently, a uniform posterior distribution is enforced. 3) A third safe zone assumes that measured kinematics of a squat motion is available, while also accounting for damage. The posterior distribution of the TF kinematics is modeled as normally distributed with a mean equal to the measured kinematics and standard deviation of 0.5 mm or 0.5° for M/L and A/P translation and I/E rotation and 1.0 mm or 1.0° for P/D translation and V/V rotation. These safe zones will be referred to as *SZ*_*D*_, *SZ*_*D&S*_, and *SZ*_*D&K*_, respectively. Further details on the definition of the posterior distributions can be found in Bartsoen et al. (2023).

For each of the native safe zones, a set family of 10,000 sets of ligament parameters is collected using BPE. We collect 10,000 sets to ensure enough data to train the post-TKA surrogate model (Section 2.3). These sets are defined within the sampling bounds given in Table 2. The analysis is performed with a feasible computational cost by using an ANN as a surrogate model of the native MSKM. The transitional Markov chain Monte Carlo (TMCMC) (Ching and Chen, 2007; Betz et al., 2016) algorithm is used to perform the BPE. For further details on the implementation, we refer to Bartsoen et al. (2023). This study determines the possible ligament properties based on experimental measurement data of the kinematics of a squat motion. The gathered sets of ligament properties are consistent with *SZ*_*D&K*_. Comparison of the set families of ligament properties between specimens allows to study the variation in properties throughout the population.

**TABLE 2:** Training bounds of the ANN for the ligament material properties.

Ligaments	*ϵ*_*r*_		*k [N]*		*Femur [mm]*		*Tibia [mm]*	
	Min	Max	Min	Max	*x*_*F*_	*y*_*F*_	*x*_*T*_	*y*_*T*_
deepMCL	−0.2	0.3	2000	9,000	[−10, 10]	[−8, 8]	[−10, 10]	
supMCL	−0.2	0.2	2000	9,000	[−10, 10]	[−8, 8]	[−10, 10]	
LCL	−0.2	0.2	2000	9,000	[−8, 8]	[−8, 8]	[−5, 5]	
ALL	−0.3	0.2	2000	9,000	[−8, 8]	[−8, 8]	[−10, 10]	
PFL	−0.3	0.2	2000	9,000	[−10, 10]	[−6, 6]		
ACL0	−0.2	0.4	4,000	10,000	[−6, 6]	[−6, 6]	[−8, 8]	[−8, 8]
ACL1	−0.2	0.4	4,000	10,000	[−6, 6]	[−6, 6]	[−8, 8]	[−8, 8]
PCL0	−0.5	0.4	4,000	12,000	[−6, 6]	[−6, 6]	[−8, 8]	[−8, 8]
PCL1	−0.5	0.4	4,000	12,000	[−6, 6]	[−6, 6]	[−8, 8]	[−8, 8]
PC	0.05	0.2	5,000	10,000				

### 2.3 TKA surrogate model

The developed TKA knee surrogate model is an ANN. This network is implemented using TensorFlow 2.4.0 (Abadi et al., 2016). This results in a network with respectively 51 and 45 input parameters for the native and post-TKA MSKM. Further information on the ANN for the native MSKM can be found in Bartsoen et al. (2023). Although, where the native ANN is trained on the entire input parameter range has been given in Table 2, this is not required for the post-TKA ANN. The post-TKA ANN can be trained on the set family satisfying *SZ*_*D*_. As *SZ*_*D&S*_ and *SZ*_*D&K*_ are subfamilies of *SZ*_*D*_, the network will also be valid for those native safe zones. The implant position parameters and flexion angle are assigned using Sobol sampling. The sampling bounds are taken at [−8,8] mm or ° with respect to the implant position consistent with mechanical alignment (Knee Planner of Materialise N.V., Leuven, Belgium).

The ANN has a fully connected architecture [45:128:256:512:256:128:64:11] with an activation function called Softplus given in Eq. 1. Further details on the training can be found in the Supplementary Material. The post-TKA network is only trained for subject 1.

$$a\left(x\right)=\log\left(\exp\left(x\right)+1\right).$$ (1)

### 2.4 Implant position optimization

To quantify the GSP of the ligament-balanced pre-operative planning, the mean (planned) implant position is optimized toward the position that results in the largest success probability given surgical precision and uncertainty in the ligament properties. The optimization is performed for the three set families of physiologically relevant ligament properties according to *SZ*_*D*_, *SZ*_*D&S*_, and *SZ*_*D&K*_. Two different types of surgical methods are evaluated, namely, RAS (Cosendey et al., 2021) and *Pr*_90%_ (Bartsoen et al., 2021). Evaluation of the objective function requires an evaluation of uncertainty caused by surgical precision and ligament properties. This is performed by applying a Monte Carlo simulation (MCS) with 4,096 samples for seven flexion angles equally divided between 20° and 120°. The number of samples is chosen based on a convergence analysis. Further details can be found in the Supplementary Material.

The samples for the MCS are taken by random sampling of normally distributed implant position parameters with a mean of 0.0 mm or ° and standard deviations given in Table 1. This set family of samples is indicated with $S_{\mathrm{MCS}}^{x=0}$ . To avoid differences between iterations due to statistical noise on the output of the MCS, $S_{\mathrm{MCS}}^{x=0}$ is constant throughout the optimization and transformed based on the mean of the implant position parameters of the current iteration. In Eq. 2, ***x*** represents the 12 DOFs of the implant position, and $S_{\mathrm{MCS}}^{x}$ represents the transformed samples.

$$S_{\mathrm{MCS}}^{x}=S_{\mathrm{MCS}}^{x=0}+x.$$ (2)

The transformed set family of samples $S_{\mathrm{MCS}}^{x}$ is combined with samples from the subject-specific set family of ligament properties, resulting in the set family of samples *S*_*MCS*_ used for estimating global uncertainty.

The optimization toward the implant position is defined as in Eq. 3, where ***x*** represents the 12 DOFs of the implant position, *S*_*MCS*_ represents the samples from the MCS, *n*_*MCS*_ is the number of samples in the MCS, *θ*_*FE*_ is the knee flexion angle (20°–120°), *ϵ* is the strain in a ligament, L is the set of all ligaments (deepMCL, supMCL, LCL, ALL, PFL, and PC), *L*_*lat*_ is the set of all lateral ligaments (LCL, ALL, and PFL), and *L*_*med*_ is the set of all medial ligaments (deepMCL and supMCL). $\epsilon_{\max}^{L_{\mathrm{lat}}}$ and $\epsilon_{\max}^{L_{\mathrm{med}}}$ are the maximal strains in *L*_*lat*_ and *L*_*med*_, respectively. $\epsilon_{\max}^{t}=6\;\%$ is the upper bound on the maximal strain in the ligaments.

$$\begin{array}{c} \underset{x}{\min}\;\frac{10}{n_{\mathrm{MCS}}}\underset{S_{\mathrm{MCS}}}{\sum }\left(u_{1}+u_{2}\right)+0.005\overset{12}{\underset{1}{\sum }}x_{i}^{2},\;\;\;\;\;\;\;\\ with\;\left\{\begin{array}{cc} u_{1}\; & =\underset{L}{\sum }\underset{\theta_{FE}:\epsilon >\epsilon_{\max}^{t}}{\sum }{\left(\epsilon -\epsilon_{\max}^{t}\right)}^{2}\\ u_{2}\; & =\underset{\theta_{FE}:\epsilon_{\max}^{L_{\mathrm{lat}}}<0}{\sum }{\left(\epsilon_{\max}^{L_{\mathrm{lat}}}\right)}^{2}+\underset{\theta_{FE}:\epsilon_{\max}^{L_{\mathrm{med}}}<0}{\sum }{\left(\epsilon_{\max}^{L_{\mathrm{med}}}\right)}^{2} \end{array}\right. \end{array}.$$ (3)

The package pymoo (Blank and Deb, 2020) is used to perform the optimization. A genetic algorithm is applied as this is a global optimization algorithm, which has the large advantage that it is unlikely to converge to a local minimum of the objective function as long as the population size is chosen large enough. Further details on the implementation can be found in the Supplementary Material.

### 2.5 Sensitivity analysis

To collect samples for the SA, the same optimization as described in Section 2.4 is performed but with constant ligament properties. This optimization is executed for 750 sets of ligament properties that were gathered with BPE using *SZ*_*D&S*_. To quantify the uncertainty caused by the implant position parameters, a quasi-Monte Carlo simulation (QMCS) is used. A QMCS uses a low-discrepancy sequence to generate the samples for the MCS. The application of a low-discrepancy sequence allows the convergence of the set of samples toward the aimed statistical distribution with a smaller number of samples compared to a random generation of samples as used with standard MCS. In this study, the Sobol sequence is applied as a low-discrepancy sequence. A convergence analysis shows that 256 samples are required. Details on the convergence analysis can be found in the Supplementary Material.

To identify the most critical parameters for the critical implant position parameters (as identified by Bartsoen et al. (2021)) and the success probability, the delta moment-independent sensitivity measure (Borgonovo, 2007) is computed. This measure is based on the difference in probability density of the model output parameter including all parameters and keeping one parameter constant. In contrast to variance-based global sensitivity measures, like Sobol indices (Sobol, 2001), the delta moment-independent measure does not rely on a single moment, for example, variance, to assess parameter sensitivity. The measure takes into account the entire input/output distribution. The SALib Python library (Herman and Usher, 2017) is applied.

A convergence analysis is performed to identify the required number of samples. A convergence measure is defined that quantifies the changes in the 10 most critical ligament parameters with respect to the ground truth 10 most critical. The ground truth set is defined based on the total number of samples gathered. For every ground truth, the critical ligament property that is not in the evaluated set of 10 critical properties, a penalty is added to the convergence measure. The size of this penalty depends on the rank (*r*) in the ground truth most critical, where the most critical parameter receives *r* = 1 and the least critical receives *r* = 10. The penalties are determined according to Eq. 4.

$$p=\frac{10.0}{r}.$$ (4)

## 3 Results

The subject-specific uncertainty in the ligament properties represented by a set family shows a similar variation for *SZ*_*D*_ and *SZ*_*D&S*_ and a slightly smaller variation for *SZ*_*D&K*_. As in Bartsoen et al. (2023), a high correlation between the reference strain and attachment sites has been identified. This correlation is nevertheless more pronounced for *SZ*_*D&K*_. Further details on the subject-specific uncertainty can be found in the Supplementary Material.

The validation accuracy of the post-TKA neural network was aimed at a 90th percentile of the absolute error (AE) below 3% for each of the ligament strains. This accuracy is minimally required compared to an optimization objective specifying the maximal strain at 6%. A total of 18,929 samples is required to achieve this accuracy objective. Details on the post-TKA neural network validation error can be found in the Supplementary Material.

The results of the implant position optimization are discussed in more detail in Section 3.1 for each of the three evaluated native safe zones and the two surgical precisions. Part 3.2 gives the results on the identification of the critical ligament properties. This section closes with an evaluation of the computational efficiency of the developed method (Section 3.3).

### 3.1 Implant position optimization

Table 3 gives the optimization objective (OO)—as computed with Eq. 3—and the GSP for the different native safe zones (*SZ*_*D*_, *SZ*_*D&S*_, and *SZ*_*D&K*_) and for two different surgical precisions (RAS (Cosendey et al., 2021) and *Pr*_90%_ (Bartsoen et al., 2021)). It can be seen that the 90% success probability of *Pr*_90%_, which solely included surgical precision, is reduced to 3.0%, 13.0%, and 13.0% for *SZ*_*D*_, *SZ*_*D&S*_, and *SZ*_*D&K*_, respectively, due to the uncertainty introduced by the ligament properties. It can be seen that the results are similar to the RAS surgical precision. *SZ*_*D&S*_ and *SZ*_*D&K*_ show similar success probabilities, but OO is, however, halved. As OO is based on the square of the difference between the ligament strain and the post-TKA safe zone, this shows a reduction in variation due to ligament properties when pre-operative squat kinematics is known.

**TABLE 3:** Pre-operative planning results with different native safe zones and surgical precisions. OO = optimization objective; GSP = global success probability.

	*SZ*_*D*_		*SZ*_*D&S*_		*SZ*_*D&K*_	
	OO	GSP	OO	GSP	OO	GSP
RAS	328.2	3.44%	131.7	12.0%	48.1	15.6%
*Pr*_90%_	329.0	3.03%	132.1	12.9%	53.6	12.9%

### 3.2 Sensitivity analysis

**FIGURE 2:**
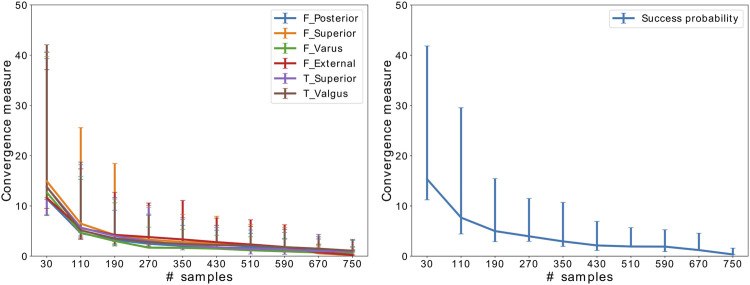
Convergence of SA for the critical implant position parameters and success probability. The error bars indicate the variation throughout 50 random samplings.

Figure 2 shows the results of the convergence analysis, implying that SA has converged after 510 samples.

**FIGURE 3:**
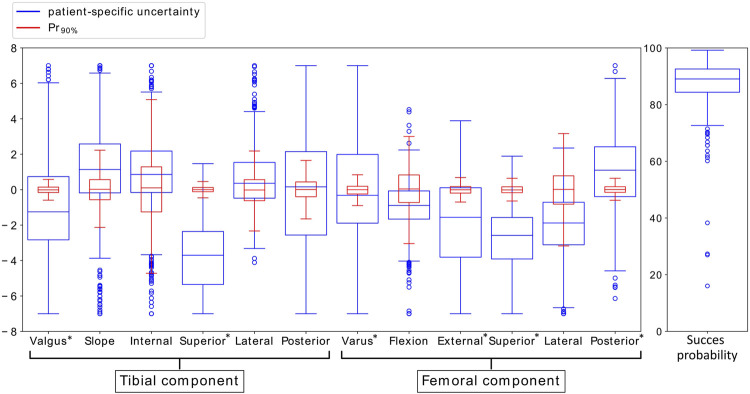
Variation in mean implant position parameters and success probability due to variation in ligament properties accounting for *SZ*_*D&S*_ (blue) and due to the surgical precision *Pr*_90%_ (red). The critical implant position parameters, identified by Bartsoen et al. (2021), are indicated with *.

Figure 3 shows the box plots of the means of the different implant position parameters for *Pr*_90%_ with variation in the ligament properties according to *SZ*_*D&S*_. This figure illustrates that the uncertainty of the critical implant position parameters (indicated with *) due to the uncertainty in the ligament properties (blue) is large compared to the required surgical error that was established in Bartsoen et al. (2021) (red). The success probability does not vary largely.

Table 4 presents the results of SA. The table gives the 10 most critical parameters, out of the 50 ligament properties, for each of the critical implant position parameters, as identified by Bartsoen et al. (2021), as well as the success probability. Each of the critical ligament properties is further divided according to their corresponding delta moment-independent sensitivity measure. The three largest measures are indicated as most critical, the next three are indicated as mid-critical, and the remaining four are indicated as least critical. Mainly, the reference strain and, to a slightly lesser extent, the attachment sites are most critical. The linear stiffness is of lesser importance. It can also be seen that for every ligament, at least one of the properties is critical.

**TABLE 4:** Ten most critical ligament properties (out of 50) for the critical implant position parameters and success probability. The 10 most critical ligament properties are further classified into the three most critical, three mid-critical, and four least critical.

	Output	Critical	Reference strain	Stiffness	Attachment	
					*Femur*	*Tibia*
Femur	A/P	Most	deepMCL and PFL		deepMCL	
		Mid	ALL and PC			ACL1
		Least			ACL0 and supMCL	ACL0 and PCL1
	P/D	Most	ALL and PFL			PCL1
		Mid	LCL		ACL0	ACL1
		Least	deepMCL	supMCL	PFL	PCL0
	V/V	Most	ALL and PFL		ACL0	
		Mid	LCL			ACL0 and PCL0
		Least			deepMCL and PFL	ACL1 and PCL1
	I/E	Most	ALL, deepMCL, and PFL			
		Mid	LCL		deepMCL	PCL1
		Least	ACL0, PC, and supMCL		ACL0	
Tibia	P/D	Most	ALL, deepMCL, and LCL			
		Mid			deepMCL and PFL	PCL1
		Least	PC and PFL		ACL0	ACL1
	V/V	Most	ALL and PFL			ACL0
		Mid	LCL		ACL0 and deepMCL	
		Least	deepMCL and PC		PFL	PCL1
	Success probability	Most	ALL, LCL, and PFL			
		Mid	deepMCL		deepMCL	PCL1
		Least		ALL	ACL0	ACL0 and ACL1

### 3.3 Computational efficiency

The optimization process to quantify GSP requires 4,096 knee model evaluations for seven flexion angles per chain, with a total of 64 offspring per generation. As each optimization requires on average 400 iterations, this results in a total of 734 million evaluations per optimization. With an evaluation time of ±4 min for the knee model directly, this analysis would be infeasible. Using the ANN as a surrogate results in an evaluation time of ±1 ms, allowing optimization in 10 days on the “AMD EPYC 7601 32-core processor.” The neural network nevertheless requires training with 17,036 training samples and 1,893 validation samples. With 12 parallel workers, this takes about 47 h to sample on “Intel Xeon CPU E5-2630.”

## 4 Discussion

We developed a ligament-balanced probabilistic planning tool that accounts for subject-specific and surgical uncertainty with a feasible computational cost. Three different native safe zones (*SZ*_*D*_, *SZ*_*D&S*_, and *SZ*_*D&K*_), representing physiological ligament strains, were evaluated along using two different surgical transfer techniques (RAS and *Pr*_90%_). Our results show that the OO values and the GSP are similar between RAS and *Pr*_90%_. It is important to note that the RAS precision quantified by Cosendey et al. (2021) is based on experimental measurements on sawbones but not in a clinical setting and documents the precision of the cuts and not the final implant position. Actual errors are thus most likely larger but are not available in the literature for all DOFs of the implant position.

With solely accounting for ligament damage, only 3% GSP can be achieved, meaning that without exact measurements of the ligament properties, the success probability is reduced by 87%. Even if an extra constraint on stability is applied, the GSP only increases to 13%. When squat kinematics are measured, the GSP does not increase further. The OO is, however, more than halved, showing that the total variation in the ligament strain is considerably reduced. Interestingly, the results show that measuring native kinematics can reduce ligament properties’ uncertainty, but that solely measuring kinematics during a squat movement is insufficient. Combining different movements, for example, laxity trials in several DOFs, could potentially offer a large enough reduction in ligament properties’ uncertainty. A device allowing this type of measurement is being studied by Pedersen et al. (2019). They developed an arthrometer that is applied in combination with a biplanar x-ray system to measure knee joint laxity in four DOFs.

It has to be highlighted that the success probabilities, as estimated in the current study, do not account for the experience of the surgeon. The success probabilities indicate the probability of success when the proposed plan would be blindly executed without further adjustments of the implant position and/or ligament releases based on the surgeon’s expertise. Consequently, in the literature, reported patient satisfaction is higher.

The results of the SA show that the variance in reference strain and attachment sites causes the small GSP, where the linear stiffness is of lesser importance. A single most critical ligament could not be identified, where even the attachment sites of the ACL and PCL are of high importance even when a PS implant is implanted. The high importance of ACL and PCL also partially explains the low success probabilities as their effect cannot be accounted for using a PS implant, where the cruciate ligaments are sacrificed. The small GSP, while imposing native squat kinematics, could also partially be explained by the use of a PS implant, where other studies have shown that it cannot entirely replicate native kinematics. Zhao et al. (2015), for example, concluded that the PS implant had an abnormal forward displacement, insufficient rollback of the lateral femoral condyle, and the tibia presented insufficient internal rotation during early flexion. Dejtiar et al. (2020), however, showed that a cruciate retaining (CR) implant is capable of approximating native kinematics. Therefore, it would be interesting to perform the same analysis with a CR implant to investigate how implant type affects the success probability.

This study needs to be considered in light of the following limitations. The conclusions are based on a single subject. The computational knee model is based on an *in vitro* squat simulation, where only the quadriceps and hamstring muscles are modeled and considered passive structures through which an external, known force is applied. A model with active muscles would require the introduction of extra patient-specific input parameters to the knee model, which would introduce extra uncertain parameters that have to be accounted for in the probabilistic knee model.

To allow clinical application of the designed pre-operative planning process in the future, the native and post-TKA safe zones need to be verified. The different safe zones are based on strain measurements and experimental damage testing in ligaments but have not been linked to patient satisfaction yet.

Another requirement to facilitate clinical application is to increase computational knee model robustness. Indeed, the current knee model formulation assumes each ligament with one strand with the exception of ACL and PCL where two strands are used. To reduce the effect of the attachment sites, each ligament could be modeled with multiple strands. As the attachment sites and reference strain are highly correlated, this approach will also decrease the influence of the reference strain. If this approach still proves insufficient to further reduce ligament-balanced implant position uncertainty, the focus of future research should be on identifying reference strain and attachment sites prior to surgery to achieve an accurate prediction. Research has been investigating *in vivo* measurement techniques to measure ligament strains. Slane et al. (2017), for example, discussed ultrasound elastography for measuring knee ligament properties *in vivo*. However, issues related to the measurement of 3D ligament movement using 2D imaging techniques are limiting accurate strain measurements. Alternatively, the pre-operative planning could be tuned intra-operatively. RAS or augmented reality (AR) systems could allow extra measurements of ligament properties through tracking of passive movements and force measurements. The motion and/or force data can be converted into an estimation of the ligament properties as shown in the current study in the estimation of the set family corresponding to *SZ*_*D&K*_. This set family identifies the ligament properties corresponding to the kinematics of a squat movement. The same procedure could be followed to quantify a set family of ligament properties corresponding to other movements and/or forces as well. As performed in the current study, the computed set family can be translated into remaining uncertainty in the ligament strains or other knee model output parameters that might be of interest.

A final step toward clinical applicability is to further decrease computational costs. Even though the use of an ANN as a surrogate for the knee model reduces the computational time of the pre-operative planning process significantly, its application in clinical practice is still infeasible as a global optimization with subject-specific and surgical uncertainty still requires several days. A possibility to further reduce this is by the application of a single loop scheme—as, for example, presented by Hong et al. (2022) through their Bayesian augmented space learning (BASL) method—where a direct prediction of the optimized mean implant position along with its remaining uncertainty would be performed. This would allow prediction of the pre-operatively planned implant position in a few milliseconds. In addition, the ANN should be trained on a patient-specific basis. Future versions of the network should therefore include patient geometry, allowing the definition of one single ANN for the pre-operative planning of individual (unseen) patients.

## 5 Conclusion

We developed a ligament-balancing probabilistic planning tool for TKA that accounts for uncertainty in ligament properties and surgical precision. Through inverse uncertainty quantification, a set family of ligament properties was identified that satisfies different physiologically relevant native safe zones. We concluded that only a GSP of 12% can be reached, meaning that without extra measurements of kinematics and/or direct measurements of ligament properties, uncertainty is too large to reduce the risk of ligament damage. A SA showed that the reference strain and to a lesser extent also the attachment sites were the most critical parameters. However, no single ligament could be identified as being the most critical parameter.

## Data Availability

The raw data supporting the conclusion of this article will be made available by the authors, without undue reservation.

## References

[B1] Abadi M. Agarwal A. Barham P. Brevdo E. Chen Z. Citro C. (2016). Tensorflow: Large-scale machine learning on heterogeneous distributed systems. arXiv preprint arXiv:1603.04467.

[B2] Bartsoen L. Faes M. G. Wesseling M. Wirix-Speetjens R. Moens D. Jonkers I. (2021). Computationally efficient optimization method to quantify the required surgical accuracy for a ligament balanced tka. IEEE Trans. Biomed. Eng. 68, 3273–3280. 10.1109/tbme.2021.3069330 33780331

[B3] Bartsoen L. Faes M. G. Andersen M. S. Wirix-Speetjens R. Moens D. Jonkers I. (2023). Bayesian parameter estimation of ligament properties based on tibio-femoral kinematics during squatting. Mech. Syst. Signal Process. 182, 109525. 10.1016/j.ymssp.2022.109525

[B4] Beidokhti H. N. Janssen D. van de Groes S. Hazrati J. Van den Boogaard T. Verdonschot N. (2017). The influence of ligament modelling strategies on the predictive capability of finite element models of the human knee joint. J. Biomech. 65, 1–11. 10.1016/j.jbiomech.2017.08.030 28917580

[B5] Betz W. Papaioannou I. Straub D. (2016). Transitional markov chain monte carlo: observations and improvements. J. Eng. Mech. 142, 04016016. 10.1061/(asce)em.1943-7889.0001066

[B6] Blank J. Deb K. (2020). pymoo: Multi-objective optimization in python. IEEE Access 8, 89497–89509. 10.1109/access.2020.2990567

[B7] Borgonovo E. (2007). A new uncertainty importance measure. Reliab. Eng. Syst. Saf. 92, 771–784. 10.1016/j.ress.2006.04.015

[B8] Bourne R. B. Chesworth B. M. Davis A. M. Mahomed N. N. Charron K. D. (2010). Patient satisfaction after total knee arthroplasty: who is satisfied and who is not? Clin. Orthop. Relat. Res. 468, 57–63. 10.1007/s11999-009-1119-9 19844772 PMC2795819

[B9] Chandrashekar N. Mansouri H. Slauterbeck J. Hashemi J. (2006). Sex-based differences in the tensile properties of the human anterior cruciate ligament. J. Biomech. 39, 2943–2950. 10.1016/j.jbiomech.2005.10.031 16387307

[B10] Ching J. Chen Y.-C. (2007). Transitional markov chain monte carlo method for bayesian model updating, model class selection, and model averaging. J. Eng. Mech. 133, 816–832. 10.1061/(asce)0733-9399(2007)133:7(816)

[B11] Cosendey K. Stanovici J. Mahlouly J. Omoumi P. Jolles B. M. Favre J. (2021). Bone cuts accuracy of a system for total knee arthroplasty including an active robotic arm. J. Clin. Med. 10, 3714. 10.3390/jcm10163714 34442008 PMC8397104

[B12] De Vloo R. Pellikaan P. Dhollander A. Vander Sloten J. (2017). Three-dimensional analysis of accuracy of component positioning in total knee arthroplasty with patient specific and conventional instruments: a randomized controlled trial. Knee 24, 1469–1477. 10.1016/j.knee.2017.08.059 28943039

[B13] Dejtiar D. L. Bartsoen L. Perez M. A. Vander Sloten J. Wirix-Speetjens R. Wesseling M. (2020). Standard cruciate-retaining total knee arthroplasty implants can reproduce NativeKinematics. Tech. rep.

[B14] Delport H. P. Vander Sloten J. Bellemans J. (2013). New possible pathways in improving outcome and patient satisfaction after tka. Acta Orthop. Belg. 79, 250–254. 23926724

[B15] Delport H. Labey L. Innocenti B. De Corte R. Vander Sloten J. Bellemans J. (2015). Restoration of constitutional alignment in tka leads to more physiological strains in the collateral ligaments. Knee Surg. Sports Traumatol. Arthrosc. 23, 2159–2169. 10.1007/s00167-014-2971-z 24705849

[B16] Freeman M. Insall J. N. Besser W. Walker P. S. Hallel T. (1977). Excision of the cruciate ligaments in total knee replacement. Clin. Orthop. Relat. Res. 126, 209–212. 10.1097/00003086-197707000-00039 598119

[B17] Guo Z. Freeman J. W. Barrett J. G. De Vita R. (2015). Quantification of strain induced damage in medial collateral ligaments. J. Biomech. Eng. 137. 10.1115/1.4030532 25955979

[B18] Hampp E. L. Chughtai M. Scholl L. Y. Sodhi N. Bhowmik-Stoker M. Jacofsky D. J. (2019). Robotic-arm assisted total knee arthroplasty demonstrated greater accuracy and precision to plan compared with manual techniques. J. Knee Surg. 32, 239–250. 10.1055/s-0038-1641729 29715696

[B19] Harner C. D. Xerogeanes J. W. Livesay G. A. Carlin G. J. Smith B. A. Kusayama T. (1995). The human posterior cruciate ligament complex: an interdisciplinary study: Ligament morphology and biomechanical evaluation. Am. J. Sports Med. 23, 736–745. 10.1177/036354659502300617 8600743

[B20] Herman J. Usher W. (2017). SALib: An open-source python library for sensitivity analysis. J. Open Source Softw. 2, 97. 10.21105/joss.00097

[B21] Hong F. Wei P. Song J. Faes M. Valdebenito M. Beer M. (2022). Combining data and physical model for probabilistic analysis: A bayesian augmented space learning perspective. J. Comput. Phys. (Preprint under review).

[B22] Kuster M. Bitschnau B. Votruba T. (2004). Influence of collateral ligament laxity on patient satisfaction after total knee arthroplasty: a comparative bilateral study. Arch. Orthop. Trauma Surg. 124, 415–417. 10.1007/s00402-004-0700-7 15156332

[B23] LaPrade R. F. Bollom T. S. Wentorf F. A. Wills N. J. Meister K. (2005). Mechanical properties of the posterolateral structures of the knee. Am. J. Sports Med. 33, 1386–1391. 10.1177/0363546504274143 16002488

[B24] Lidgren L. Robertsson O. W-Dahl A. (2004). The Swedish knee arthroplasty register: Annual report 2004. Lund: Lund University Hospital.

[B25] Nam D. Nunley R. Barrack R. (2014). Patient dissatisfaction following total knee replacement: a growing concern? Bone Jt. J. 96, 96–100. 10.1302/0301-620x.96b11.34152 25381418

[B26] Noble P. C. Gordon M. J. Weiss J. M. Reddix R. N. Conditt M. A. Mathis K. B. (2005). Does total knee replacement restore normal knee function? Clin. Orthop. Relat. Res. 431, 157–165. 10.1097/01.blo.0000150130.03519.fb 15685070

[B27] Pedersen D. Vanheule V. Wirix-Speetjens R. Taylan O. Delport H. P. Scheys L. (2019). A novel non-invasive method for measuring knee joint laxity in four dof: *In vitro* proof-of-concept and validation. J. Biomech. 82, 62–69. 10.1016/j.jbiomech.2018.10.016 30384999

[B28] Pianigiani S. Croce D. D’Aiuto M. Pascale W. Innocenti B. (2017). Sensitivity analysis of the material properties of different soft-tissues: implications for a subject-specific knee arthroplasty. Muscle Ligaments Tendons J. 7, 546. 10.32098/mltj.04.2017.09 PMC5908331 29721456

[B29] Provenzano P. P. Heisey D. Hayashi K. Lakes R. Vanderby R. Jr (2002). Subfailure damage in ligament: a structural and cellular evaluation. J. Appl. Physiol. 92, 362–371. 10.1152/jappl.2002.92.1.362 11744679

[B30] Robinson J. R. Bull A. M. Amis A. A. (2005). Structural properties of the medial collateral ligament complex of the human knee. J. Biomech. 38, 1067–1074. 10.1016/j.jbiomech.2004.05.034 15797588

[B31] Sharkey P. F. Lichstein P. M. Shen C. Tokarski A. T. Parvizi J. (2014). Why are total knee arthroplasties failing today—has anything changed after 10 years? J. Arthroplasty 29, 1774–1778. 10.1016/j.arth.2013.07.024 25007726

[B32] Skipper Andersen M. De Zee M. Damsgaard M. Nolte D. Rasmussen J. (2017). Introduction to force-dependent kinematics: theory and application to mandible modeling. J. Biomech. Eng. 139. 10.1115/1.4037100 28639682

[B33] Slane L. C. Slane J. A. D’hooge J. Scheys L. (2017). The challenges of measuring *in vivo* knee collateral ligament strains using ultrasound. J. Biomech. 61, 258–262. 10.1016/j.jbiomech.2017.07.020 28802742 PMC5581255

[B34] Smith C. R. Vignos M. F. Lenhart R. L. Kaiser J. Thelen D. G. (2016). The influence of component alignment and ligament properties on tibiofemoral contact forces in total knee replacement. J. Biomech. Eng. 138, 021017. 10.1115/1.4032464 26769446 PMC4844247

[B35] Sobol I. M. (2001). Global sensitivity indices for nonlinear mathematical models and their monte carlo estimates. Math. Comput. Simul. 55, 271–280. 10.1016/s0378-4754(00)00270-6

[B36] Sugita T. Amis A. A. (2001). Anatomic and biomechanical study of the lateral collateral and popliteofibular ligaments. Am. J. Sports Med. 29, 466–472. 10.1177/03635465010290041501 11476388

[B37] Trent P. S. Walker P. S. Wolf B. (1976). Ligament length patterns, strength, and rotational axes of the knee joint. Clin. Orthop. Relat. Res. 117, 263–270. 10.1097/00003086-197606000-00034 1277674

[B38] Twiggs J. G. Wakelin E. A. Roe J. P. Dickison D. M. Fritsch B. A. Miles B. P. (2018). Patient-specific simulated dynamics after total knee arthroplasty correlate with patient-reported outcomes. J. Arthroplasty 33, 2843–2850. 10.1016/j.arth.2018.04.035 29807792

[B39] Vanheule V. Delport H. P. Andersen M. S. Scheys L. Wirix-Speetjens R. Jonkers I. (2017). Evaluation of predicted knee function for component malrotation in total knee arthroplasty. Med. Eng. Phys. 40, 56–64. 10.1016/j.medengphy.2016.12.001 27989384

[B40] Victor J. Van Glabbeek F. Vander Sloten J. Parizel P. M. Somville J. Bellemans J. (2009a). An experimental model for kinematic analysis of the knee. J. Bone Jt. Surg. 91, 150–163. 10.2106/jbjs.i.00498 19884423

[B41] Victor J. Wong P. Witvrouw E. Vander Sloten J. Bellemans J. (2009b). How isometric are the medial patellofemoral, superficial medial collateral, and lateral collateral ligaments of the knee? Am. J. Sports Med. 37, 2028–2036. 10.1177/0363546509337407 19589921

[B42] Woo S. L.-Y. Hollis J. M. Adams D. J. Lyon R. M. Takai S. (1991). Tensile properties of the human femur-anterior cruciate ligament-tibia complex: the effects of specimen age and orientation. Am. J. Sports Med. 19, 217–225. 10.1177/036354659101900303 1867330

[B43] Zhao Z.-X. Wen L. Qu T.-B. Hou L.-L. Xiang D. Bin J. (2015). Kinematic analysis of a posterior-stabilized knee prosthesis. Chin. Med. J. 128, 216–221. 10.4103/0366-6999.149205 25591565 PMC4837841

