# Supplementary material for: Probabilistic planning for ligament-balanced TKA—Identification of critical ligament properties
Source: Front Bioeng Biotechnol. 2022 Nov 17;10:930724. doi: 10.3389/fbioe.2022.930724 (PMC9713239; doi:10.3389/fbioe.2022.930724)
Supplement: Supplementary file 1 [file DataSheet1.PDF]

Supplementary Material

1 LIGAMENT MATERIAL BEHAVIOUR

The force-strain relationship is defined in eq. (S1), where F is the force, ϵ is the strain, k is the ligament stiffness and ϵ_l is an experimentally fitted parameter equal to 0.03. The strain ϵ can be computed through eq. (S2), where L_r and ϵ_r are respectively the strand length and strain at reference (extended) position.

$$F = \begin{cases} 0 & if \quad \epsilon < 0 \\ \frac{1}{4}\frac{k\epsilon^2}{\epsilon_l} & if \quad 0 \leq \epsilon \leq 2\epsilon_l \\ k(\epsilon - \epsilon_l) & if \quad \epsilon > 2\epsilon_l \end{cases} \tag{S1}$$

$$\epsilon = \frac{(1+\epsilon_r)L - L_r}{L_r} \tag{S2}$$

2 KNEE SURROGATE MODEL TRAINING

The ANN is trained using the Adam optimizer (Kingma and Ba, 2014). The learning rate decays upon plateau of the loss and starts at 0.001. The loss function that is used is the Huber loss given in eq. (S3). This function behaves like mean absolute error (MAE) for large errors and like MSE for small errors. The parameter d is equal to 0.02. To prevent overtraining, early stopping is applied with restoration of the weights corresponding to the smallest validation loss along with L2 regularization.

$$\begin{aligned} x &= y_{true} - y_{predicted} \\ loss &= \begin{cases} 0.5\ x^2 & if\ |x| \leq d \\ 0.5\ d^2 + d\ (|x| - d) & if\ |x| > d \end{cases} \end{aligned} \tag{S3}$$

3 CONVERGENCE ANALYSIS IMPLANT POSITION OPTIMIZATION

The number of samples required for the quantification of uncertainty in the probabilistic optimization of the planned implant position is selected through a convergence analysis. For every number of samples indicated on the x-axis of fig. S1, 50 random samplings are performed. The figure indicates the mean and variation within those samplings with respect to the ground truth (10 000 samples). Fig. S1 shows that convergence has occurred at 4 096.

4 OPTIMIZATION ALGORITHM

To perform the optimization of the implant position, a genetic algorithm is applied. These algorithms consists out of a sequence of three operations, namely selection, crossover and mutation. The initial population is sampled using the Sobol sequence. The selection is performed using Tournament selection. The crossover algorithm is Differential evolution with probability $p = 0.95$. The mutation algorithm is Polynomial mutation with probability $p = 0.1$ and $D = 20$. The parameter D regulates the width of the exponential probability distribution. The population size was chosen at 256 and the offspring amount is equal to 64. The review of Piotrowski (2017) shows that a population size lower than 50 individuals is rarely recommended and that the optimal population and offspring sizes are highly problem dependent. As

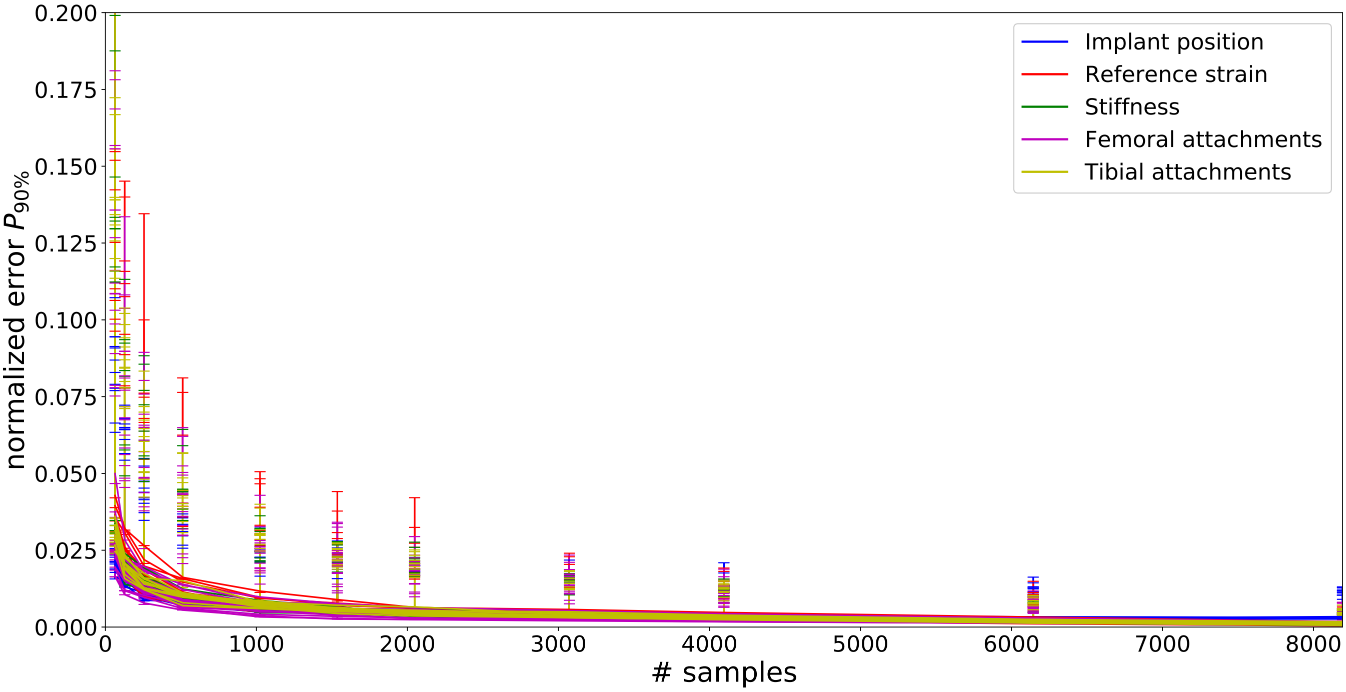

Figure S1. Monte Carlo simulation - Convergence of the 90th percentile ($P_{90\%}$). The error bars indicate the variation throughout 50 random samplings.

a consequence, they were selected through trial-and-error varying with powers of 2 allowing a trade-off between solution accuracy and convergence speed.

5 CONVERGENCE ANALYSIS SA

To identify the critical ligament properties, a sensitivity analysis (SA) is performed. To quantify uncertainty caused by the implant position parameters, a quasi Monte Carlo Simulation (QMCS) is used. The study of Burhenne et al. (2011) shows that application of the low discrepancy Sobol sequence is appropriate to perform a QMCS. As illustrated in figure S2 and mentioned by the study of Burhenne et al. (2011), powers of 2 samples have to be used to result in the same sets for al parameters. Figure S2 gives the error on the 90th ($P_{90\%}$), 95th ($P_{95\%}$) and 99th ($P_{99\%}$) percentiles for different numbers of samples. This is performed for a set of 12 parameters as the implant position has 12 degrees of freedom (DOFs). Figure S2**A** gives the results for powers of 2, figure S2**B** gives the same results but for 1.1 times the powers of 2. As can be seen convergence is more variable with non-powers of 2. Convergence occurs at 256 samples.

6 SUBJECT-SPECIFIC UNCERTAINTY

Table S1 gives the difference between the 5th percentile ($P_{5\%}$) and $P_{95\%}$ of the variation of the input parameters for each of the native safe zones. We can see that absolute variation is similar for SZ_D and $SZ_{D\&S}$ and slightly smaller for $SZ_{D\&K}$.

The coefficient of determination (R^2) of the linear regression analysis are given in table S2. It can be seen that the reference strain and the attachment sites are highly correlated while the linear stiffness and reference strain are not, neither are the linear stiffness and attachment sites. This is consistent throughout the three safe zones, but are more pronounced with $SZ_{D\&K}$.

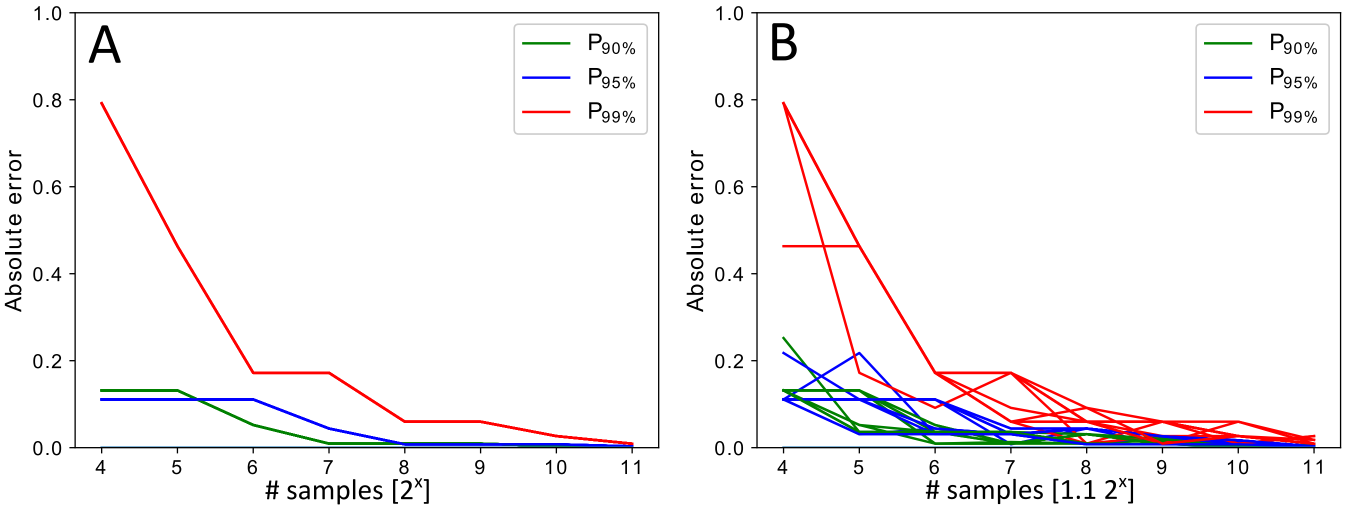

Figure S2. QMCS - Sobol sequence convergence. Convergence of the $P_{90\%}$, $P_{95\%}$ and $P_{99\%}$ percentiles. (**A**) 2^x, (**B**) $1.1 \cdot 2^x$

Table S1. Difference between $P_{5\%}$ and $P_{95\%}$ of the variation of the input parameters. The absolute variation is given for each of the three combinations of ligament parameters.

**Ligaments**	ϵ_r			*k [N]*			*Attachments [mm]*		
	SZ_D	$SZ_{D\&S}$	$SZ_{D\&K}$	SZ_D	$SZ_{D\&S}$	$SZ_{D\&K}$	SZ_D	$SZ_{D\&S}$	$SZ_{D\&K}$
deepMCL	0.33	0.34	0.25	6268	6193	4267	16.4	15.4	11.7
supMCL	0.21	0.18	0.21	6286	6228	5943	16.6	15.8	14.0
LCL	0.28	0.26	0.15	6277	6040	4869	12.5	12.2	9.4
ALL	0.25	0.28	0.19	6152	5982	5763	14.8	13.4	11.2
PFL	0.33	0.35	0.19	6257	6054	5863	14.2	14.2	9.3
ACL0	0.37	0.35	0.15	5379	5264	4600	13.9	13.9	10.2
ACL1	0.4	0.44	0.29	5396	5319	5293	14.3	14.0	13.2
PCL0	0.51	0.55	0.48	7156	7110	6759	14.7	14.2	12.7
PCL1	0.55	0.54	0.34	7120	7075	6321	14.9	14.6	10.8
PC	0.11	0.11	0.08	4463	4379	4415			

7 TKA SURROGATE MODEL

The accuracy of the network is given in table S3 for subject 1. The 90 % absolute error (AE) was aimed to be below 3 % for the ligament strains and below 2 mm or ° for tibio-femoral (TF) kinematics. It can be seen that this is achieved for each output. The strain in the posterior cruciate ligament (PCL) has the largest error. This is to be expected as it is the ligament with the largest variance in strain throughout the sampling space as it does not have the largest normalized MAE (nMAE) but it has the largest MAE and 90 % AE. The number of samples required for training was 18 929 samples.

Table S2. Results of linear regression analysis. R^2 is given for each of the three combinations of ligament parameters.

**Ligaments**	ϵ_r - *Attachments*			k - *Attachments*			k - ϵ_r		
	SZ_D	$SZ_{D\&S}$	$SZ_{D\&K}$	SZ_D	$SZ_{D\&S}$	$SZ_{D\&K}$	SZ_D	$SZ_{D\&S}$	$SZ_{D\&K}$
deepMCL	0.82	0.8	0.89	0.0	0.01	0.02	0.0	0.01	0.0
supMCL	0.82	0.83	0.85	0.0	0.01	0.0	0.0	0.01	0.01
LCL	0.55	0.19	0.72	0.01	0.03	0.12	0.0	0.01	0.03
ALL	0.3	0.12	0.88	0.01	0.03	0.0	0.01	0.01	0.01
PFL	0.34	0.07	0.91	0.01	0.0	0.03	0.01	0.01	0.01
ACL0	0.12	0.13	0.39	0.01	0.01	0.04	0.05	0.04	0.02
ACL1	0.09	0.09	0.13	0.01	0.03	0.04	0.03	0.06	0.0
PCL0	0.27	0.41	0.19	0.0	0.02	0.02	0.0	0.0	0.01
PCL1	0.37	0.21	0.67	0.0	0.01	0.11	0.0	0.0	0.0
PC							0.0	0.0	0.0

Table S3. Accuracy of artificial neural network for all outputs, with nMAE the mean absolute error with the outputs normalized between [0, 1] and 90 % AE the 90th percentile of the absolute error. The 90 % AE was aimed to be below 3 % for the ligament strains and below 2 mm or ° for TF kinematics.

	**Output**	**nMAE [%]**	**MAE [mm or °]**	**90 % AE [mm or °]**
**Kinematics**	M/L	0.52	0.20	0.42
	A/P	0.83	0.41	0.89
	P/D	0.25	0.15	0.31
	V/V	0.68	0.25	0.52
	I/E	1.15	0.76	1.57
**Ligament strain**	deepMCL	0.55	0.75	1.57
	supMCL	0.52	0.35	0.70
	LCL	0.42	0.50	1.04
	ALL	0.63	0.84	1.76
	PFL	0.50	0.66	1.35
	PC	0.50	0.57	1.17
